# Automated Quality-Controlled Cardiovascular Magnetic Resonance Pericardial Fat Quantification Using a Convolutional Neural Network in the UK Biobank

**DOI:** 10.3389/fcvm.2021.677574

**Published:** 2021-07-07

**Authors:** Andrew Bard, Zahra Raisi-Estabragh, Maddalena Ardissino, Aaron Mark Lee, Francesca Pugliese, Damini Dey, Sandip Sarkar, Patricia B. Munroe, Stefan Neubauer, Nicholas C. Harvey, Steffen E. Petersen

**Affiliations:** ^1^William Harvey Research Institute, National Institute for Health Research (NIHR) Barts Biomedical Research Centre, Queen Mary University of London, Charterhouse Square, London, United Kingdom; ^2^St Bartholomew's Hospital, Barts Health National Health Service (NHS) Trust, London, United Kingdom; ^3^Faculty of Medicine, Imperial College London, London, United Kingdom; ^4^Biomedical Imaging Research Institute, Cedars-Sinai Medical Centre, Los Angeles, CA, United States; ^5^Division of Cardiovascular Medicine, Radcliffe Department of Medicine, University of Oxford, National Institute for Health Research Oxford Biomedical Research Centre, Oxford University Hospitals NHS Foundation Trust, Oxford, United Kingdom; ^6^Medical Research Council (MRC) Lifecourse Epidemiology Unit, University of Southampton, Southampton, United Kingdom; ^7^National Institute for Health Research (NIHR) Southampton Biomedical Research Centre, University Hospital Southampton National Health Service (NHS) Foundation Trust, University of Southampton, Southampton, United Kingdom

**Keywords:** cardiovascular magnetic resonance, pericardial fat, epicardial fat, obesity, automated image analysis, neural network, machine learning

## Abstract

**Background:** Pericardial adipose tissue (PAT) may represent a novel risk marker for cardiovascular disease. However, absence of rapid radiation-free PAT quantification methods has precluded its examination in large cohorts.

**Objectives:** We developed a fully automated quality-controlled tool for cardiovascular magnetic resonance (CMR) PAT quantification in the UK Biobank (UKB).

**Methods:** Image analysis comprised contouring an en-bloc PAT area on four-chamber cine images. We created a ground truth manual analysis dataset randomly split into training and test sets. We built a neural network for automated segmentation using a Multi-residual U-net architecture with incorporation of permanently active dropout layers to facilitate quality control of the model's output using Monte Carlo sampling. We developed an in-built quality control feature, which presents predicted Dice scores. We evaluated model performance against the test set (*n* = 87), the whole UKB Imaging cohort (*n* = 45,519), and an external dataset (*n* = 103). In an independent dataset, we compared automated CMR and cardiac computed tomography (CCT) PAT quantification. Finally, we tested association of CMR PAT with diabetes in the UKB (*n* = 42,928).

**Results:** Agreement between automated and manual segmentations in the test set was almost identical to inter-observer variability (mean Dice score = 0.8). The quality control method predicted individual Dice scores with Pearson *r* = 0.75. Model performance remained high in the whole UKB Imaging cohort and in the external dataset, with medium–good quality segmentation in 94.3% (mean Dice score = 0.77) and 94.4% (mean Dice score = 0.78), respectively. There was high correlation between CMR and CCT PAT measures (Pearson *r* = 0.72, *p*-value 5.3 ×10^−18^). Larger CMR PAT area was associated with significantly greater odds of diabetes independent of age, sex, and body mass index.

**Conclusions:** We present a novel fully automated method for CMR PAT quantification with good model performance on independent and external datasets, high correlation with reference standard CCT PAT measurement, and expected clinical associations with diabetes.

## Introduction

Pericardial adipose tissue (PAT), which surrounds the surface of the heart and adventitia of the coronary arteries, has been linked to a range of important cardiovascular and metabolic conditions, including atrial fibrillation (1), diabetes (2), and coronary artery disease (3). These relationships appear independent of subcutaneous fat, total body weight, and classical cardiovascular risk factors (4), suggesting distinct biological significance of PAT. Indeed, it has been proposed that adipocytokines and proinflammatory markers secreted by metabolically active PAT may act as mediators for these associations through promotion of a milieu for disease development at both local and systemic levels (5, 6). Thus, PAT may provide novel insights into disease processes and has a potential role as a marker of cardiovascular risk.

Technical difficulties in quantification of PAT in an efficient and radiation-free manner have precluded its systematic study in large cohorts. While cardiac computed tomography (CCT) PAT quantification is well-established (3, 7–9), exposure of large population cohorts to ionizing radiation is not ethically permissible (10). Cardiovascular magnetic resonance (CMR) is the reference imaging modality for assessment of cardiac structure and function and has been used in several large population studies, including the Multi-ethnic Study of Atherosclerosis (11), the Framingham Heart Study (12), and the UK Biobank (UKB) (13). Thus, CMR PAT quantification would have high utility for research, with potential for translation into clinical care; however, existing methods require dedicated acquisitions and, often, arduous manual image analysis (14, 15), limiting their applicability to large datasets with standard sequence acquisitions.

We present a novel fully automated method for PAT quantification using standard-of-care CMR images with in-built quality control (QC) developed and tested in the UKB. We test the correlation of this CMR PAT metric with reference standard CCT PAT quantification in an external dataset and investigate clinical validity through consideration of associations with diabetes in UKB. Reporting in this paper is in accordance with relevant aspects of the Proposed Requirements for Cardiovascular Imaging-Related Machine Learning Evaluation (PRIME) guidance (16).

## Materials and Methods

### Setting and Study Population

The UKB incorporates data from over half a million participants recruited between 2006 and 2010 from across the UK. Individuals aged 40–69 years old were identified through National Health Service (NHS) registers and requested to participate *via* postal invites. There was detailed baseline characterization of participant demographics, lifestyle, and medical history (17). The UKB protocol is publicly available (18). The UKB Imaging Study, which includes detailed CMR imaging, aims to scan 100,000 of the original participants (approximately 50,000 completed, June 2021) (19).

### CMR Image Acquisition

CMR imaging was with 1.5-T scanners (MAGNETOM Aera, Syngo Platform VD13A, Siemens Healthcare, Erlangen, Germany) using a standardized acquisition protocol, which is detailed elsewhere (13). Cardiac function was assessed using balanced steady state free precession cine sequences with standard long-axis cuts and a complete short-axis stack. No signal or image filtering was applied, with the exception of distortion correction.

### Standard Operating Procedure for PAT Segmentation

**Figure 1:**
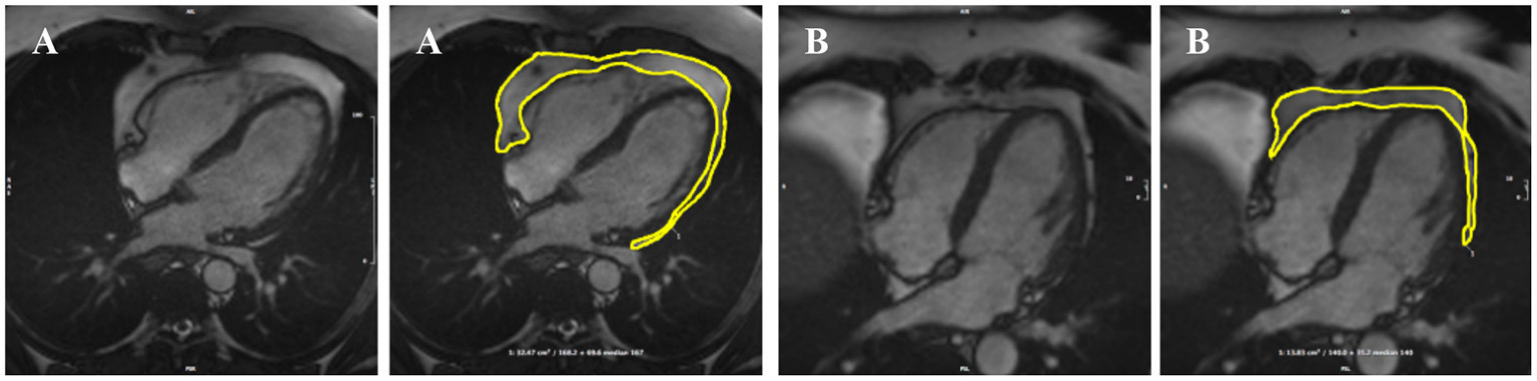
Two examples of PAT contoured in end-diastole on four-chamber bSSFP cine-CMR, performed using CVI^42®^ software according to the established SOP. A single contour was drawn to select areas of high signal intensity adjacent to the epicardial surface of the left and right ventricles, resulting in output of an area measure **(A)**. Areas of high signal intensity over the liver were not included in the PAT measure as this almost always represents adipose tissue below the diaphram **(B)**. bSSFP, balanced steady state free precession; CMR, cardiovascular magnetic resonance; PAT, pericardial adipose tissue; SOP, standard operating procedure. Images reproduced with permission of UK Biobank.

The analysis protocol comprised segmentation of an en bloc PAT area from standard four-chamber cine images (single 2D slice), a universal component of standard CMR studies and one that demonstrates less variability in cut plane positioning compared to other acquisitions (e.g., short axis slices). For consistency, we measured PAT at phase 1 of the imaging cycle (approximately end-diastole). A single contour was drawn to select areas of high signal intensity adjacent to the epicardial surface of the left and right ventricular myocardium, resulting in output of an area measure in cm^2^ (Figure 1). Areas of high signal intensity over the liver were not included, as this almost always represents adipose tissue below the diaphragm (Figure 1B).

### Creation of a Ground Truth Manual Segmentation Dataset

We selected 500 random UKB participants with record of an imaging center visit using the random number generator package in R. We excluded participants with missing (*n* = 45) or inadequate quality (*n* = 23) CMR images. PAT contours were manually drawn for the remaining 432 participants. For the purposes of model training and evaluation, the sample was randomly split into training (*n* = 345) and test (*n* = 87) sets. Image analysis was performed blind to participant details using CVI^42®^ post-processing software (Version 5.11, Circle Cardiovascular Imaging Inc., Calgary, Canada). Contours were drawn by AB and cross-checked by ZRE.

### Neural Network Architecture

As the size of PAT does not alter during the cardiac cycle, it may be quantified on static images, without consideration to cardiac motion. Thus, PAT quantification can be framed as a simple foreground-segmentation problem using a single frame per experimental subject, from which the area it occupies can be extracted. The task is to predict whether individual pixels represent a point of interest (i.e., within PAT) or are a part of the background.

**Figure 2:**
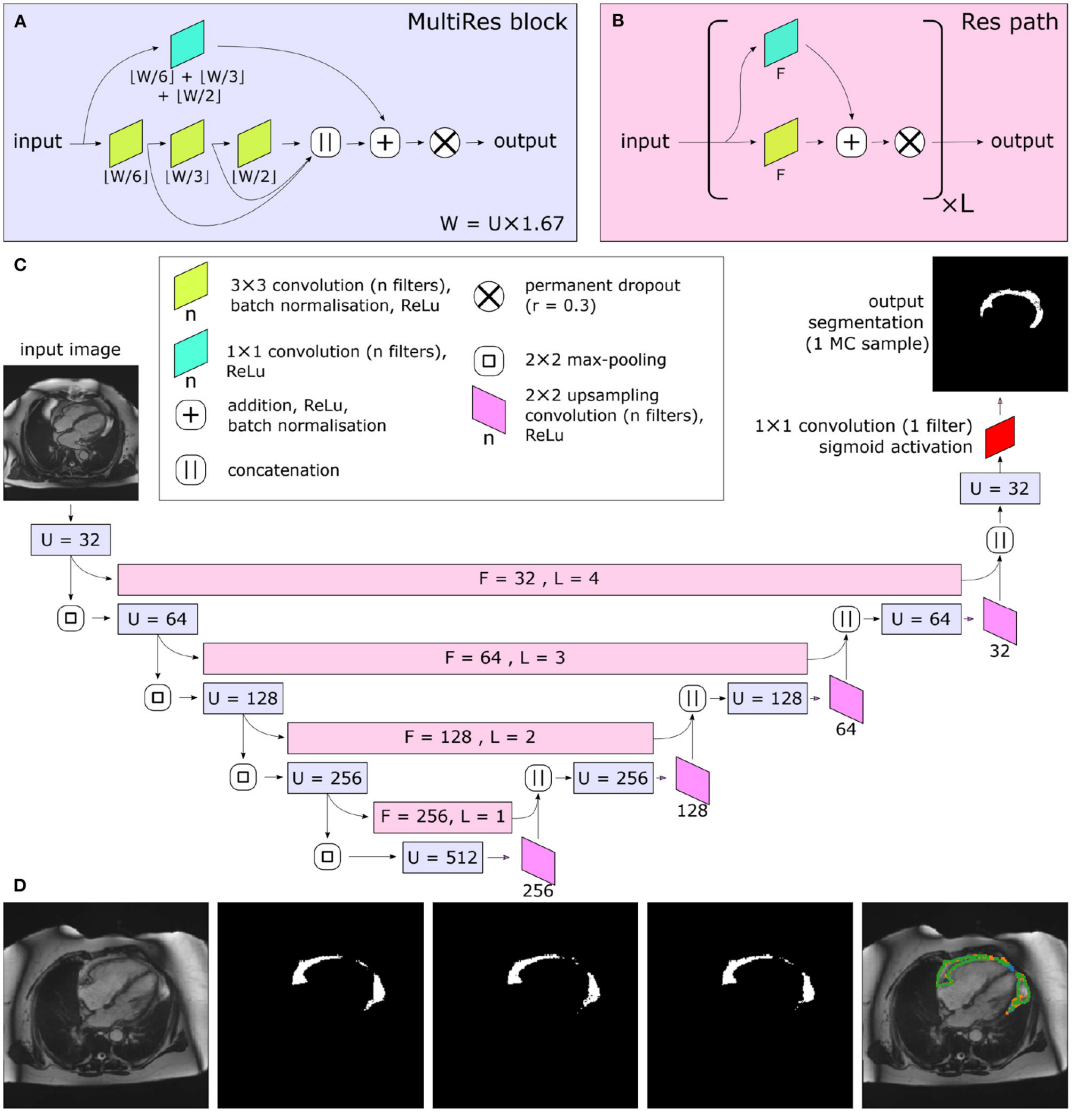
Central illustration. Summary of model architecture used in the present study. The MultiRes blocks **(A)** form the encoder and decoder arms of the network. The number of filters used throughout the different components of the block is parameterized by U. The encoder and decoder arms are joined by Res paths **(B)**, which are parameterized by F and L. They are formed of L repeating units, and their convolutional components each have F filters. The complete network is shown in **(C)**. In **(C)**, the colors indicate the placement of MultiRes blocks **(A)** and Res paths **(B)**, while the hyperparameters used in each instance of the blocks are indicated as overlaid text. Because of the permanently active dropout components, each prediction the network makes is equivalent to a Monte Carlo (MC) sample. **(D)** shows three such samples drawn based on the same input image. Note the disagreement at the edges of the segmented regions, particularly clear as shown on the overlay (far right). Images reproduced with permission of UK Biobank.

Great progress has been made with automated medical image segmentation using fully convolutional neural networks (20, 21), particularly using encoder–decoder architectures (22). We developed a neural network using a Multi-residual U-net (MultiResUNet) base architecture (23) with the incorporation of a permanently active dropout layer (24) at the end of each multi-residual block (Figure 2). The best trade-off between overall segmentation accuracy and prediction of that accuracy was obtained with a dropout rate *r* = 0.3, which we found to be optimal for model performance. This was selected as the largest *r* value at which the segmentation quality was not statistically reduced relative to a non-stochastic network (Supplementary Figure 1). To incorporate a measure of uncertainty that can be used for QC, we used permanently active dropout layers to add a stochastic component to their network outputs, meaning that multiple Monte Carlo (MC) samples can be drawn for any given input (24). This MC sampling from a stochastic neural network generates *N* samples of predicted probability maps {*P*_1_...*P*_*N*_}, from which thresholding at 0.5 can generate Boolean segmentation maps {*S*_1_...*S*_*N*_}. For our foreground detection problem, the final segmentation *S* for each voxel (*x*) is defined by thresholding the voxelwise mean of *S*:

$$S(x)=\{\begin{array}{l} 1\frac{\sum_{i=1}^{N}S_{i}(x)}{N}\geq 0.5\\ 0\;otherwise \end{array}$$

### Network Implementation

The neural network was implemented and trained using the TensorFlow 2.0 Python API (25), software available from https://www.tensorflow.org. A combination of resampling images to enforce uniform resolution, robust data augmentation, and intensity normalization has previously been shown to increase the generalizability of segmentation networks (26). Inspired by this approach, all image data were first resampled to a uniform resolution (1.82 ×1.82 mm pixel spacing) and cropped/padded to a size of 208 ×208 pixels, including the test set. During training, data were augmented with rotation (up to 25°), altered resolution (resizing of up to 20%), random shearing up to 20%, and random panning of up to 25% of the image dimension. All data augmentation was performed on-the-fly, meaning that each complete training epoch utilized a different set of images. All images were normalized such that their pixel intensity range was between 0 and 1.

Training proceeded for a maximum of 300 complete epochs on a NVIDIA Tesla M40, using a batch size of eight images. The loss function used was the binary cross-entropy, and this was optimized using the Adam optimizer (27), with an initial learning rate of 0.01, β_1_ = 0.9, β_2_ = 0.999. The learning rate was decreased by a factor of 0.3 if, for 10 consecutive epochs, the loss was not decreasing. If 20 epochs elapsed with no decrease in the loss function, training was ceased and the weights yielding the lowest loss were restored.

### Metrics for Assessment of Segmentation Agreement, Inter-observer Variability, and Model Performance

We evaluated agreement between manual segmentations of different expert observers (AB, ZRE, and SEP) and between manual and automated segmentation. When multiple MC samples are drawn from the stochastic neural network, their level of agreement is correlated with the quality of the consensus segmentation (24). We expressed level of segmentation agreement using metrics based on the well-known intersection-over-union (or Jacard Index) and the Dice score. Thus, we used four metrics for agreement between segmentations: the Dice score, Intersection-over-Union (IoU) metrics for overlap, the mean contour distance, and the symmetric Hausdorff distance (compares the closeness of foreground voxels borders). Both of the overlap metrics are bounded between 0 and 1, with 0 representing no overlap and 1 representing perfect overlap. For both of the distance metrics, lower distances represent closer agreement between segmentation results. In line with previous literature pertaining to QC (24, 28), we classified segmentation accuracy as poor, medium, or good based on Dice scores of <0.6, 0.6–0.8, and ≥0.8 respectively.

### Correlation of Automated CMR PAT Quantification With CCT Measured PAT

We tested the correlation of our derived CMR PAT measurement with established CCT PAT measurements. We utilized the Barts Health NHS Trust local sub-study, from the EValuation of INtegrated Cardiac Imaging for the Detection and Characterization of Ischaemic Heart Disease (EVINCI) dataset (29), a clinical trial dataset including *n* = 109 participants with paired CMR and CCT imaging performed within a maximum interval of 37 days. We used the QFAT software (version 2.0, Cedars-Sinai Medical Center) for CCT PAT quantification (7, 8), which segments and quantifies epicardial and thoracic fat from non-contrast calcium scoring CCT. We utilized deep learning-based contouring with no manual adjustment. Voxels containing thoracic fat deposits were defined as those with a radiodensity of between −190 and −30 Hounsfield units. The total PAT volume was measured in cm^3^. For the CMR analysis, we used our automated pipeline: four-chamber cine images were resampled to a resolution of 1.82 ×1.82 mm, padded/cropped to 208 ×208 pixels, normalized to have pixel intensities ranging between 0 and 1, and our stochastic network segmentation and QC applied with *N* = 15. Finally, the segmented area was extracted as the mean foreground area of the MC samples. Thus, we were able to test the performance of our automated image analysis pipeline on CMR studies within the EVINCII cohort and also to assess the correlation between these measures and CCT PAT quantification.

### Association With Diabetes

Given the established association between diabetes and increased PAT, we tested the clinical validity of our PAT measures through consideration of associations with this condition. We applied our automated CMR PAT analysis tool to the entire UKB Imaging cohort for whom adequate imaging was available (*n* = 42,928). Diabetes was coded based on self-report of the diagnosis, self-reported use of “medication for diabetes,” or serum glycosylated hemoglobin >48 mmol/mol. We tested the association of PAT area with diabetes status in multivariable logistic regression models with adjustment for age, sex, and body mass index (BMI). We present the results as odds ratio associated with a 10 cm^2^ increase in PAT with corresponding 95% confidence intervals and *p*-values.

## Results

### Model Training

We trained a MultiResUNet (23) with a Bayesian modification, such that multiple MC samples are drawn for each input, in order to perform QC and derive measures of uncertainty (24). Within this context, there is one hyperparameter that must be optimized after model training—that of the number (*N*) of MC samples drawn when segmenting an unseen image. When multiple MC samples are drawn during segmentation, they can be summarized in a number of ways. Firstly, they can be used to produce a single “best-guess” segmentation, *via* a simple voxel-wise voting procedure. It is expected that drawing more samples from a well-trained network will increase its accuracy, but with diminishing returns. Where the area of “foreground” pixels is particularly of interest (as in this use case, quantifying the area of PAT), we can report the mean and standard deviation of the areas across the *N* samples, which can be used for propagating uncertainty in downstream calculations. *N* was set to 15 for all further work, for the following reasons: Comparisons of segmentation accuracy with a deterministic neural network showed that consistent with prior work (24), there was no sacrifice in segmentation quality by using a stochastic network relative to a deterministic one when N was set to an appropriate level (Supplementary Figure 1A). Additionally, increasing N beyond 15 gave very little extra segmentation accuracy (Supplementary Figure 1A) or estimated standard deviation of area.

There are a number of different metrics that were proposed as correlates of final segmentation accuracy; however, it was concluded that, of these, both the most conceptually convenient and the most easily interpretable are those corresponding to often-used segmentation accuracy metrics—the Dice score and the IoU of the MC samples (24). We tested calculation of both IoU and Dice score globally or mean pairwise over the MC samples, finding that the best predictor of true segmentation accuracy was the mean pairwise Dice score between the MC samples, assessed on quantitative measures of agreement with the true Dice score of the test set (Supplementary Figure 2). Further details, as well as relevant equations, are detailed in the Supplementary Material.

### Evaluation of Automated CMR PAT Segmentation Model Performance

**Figure 3:**
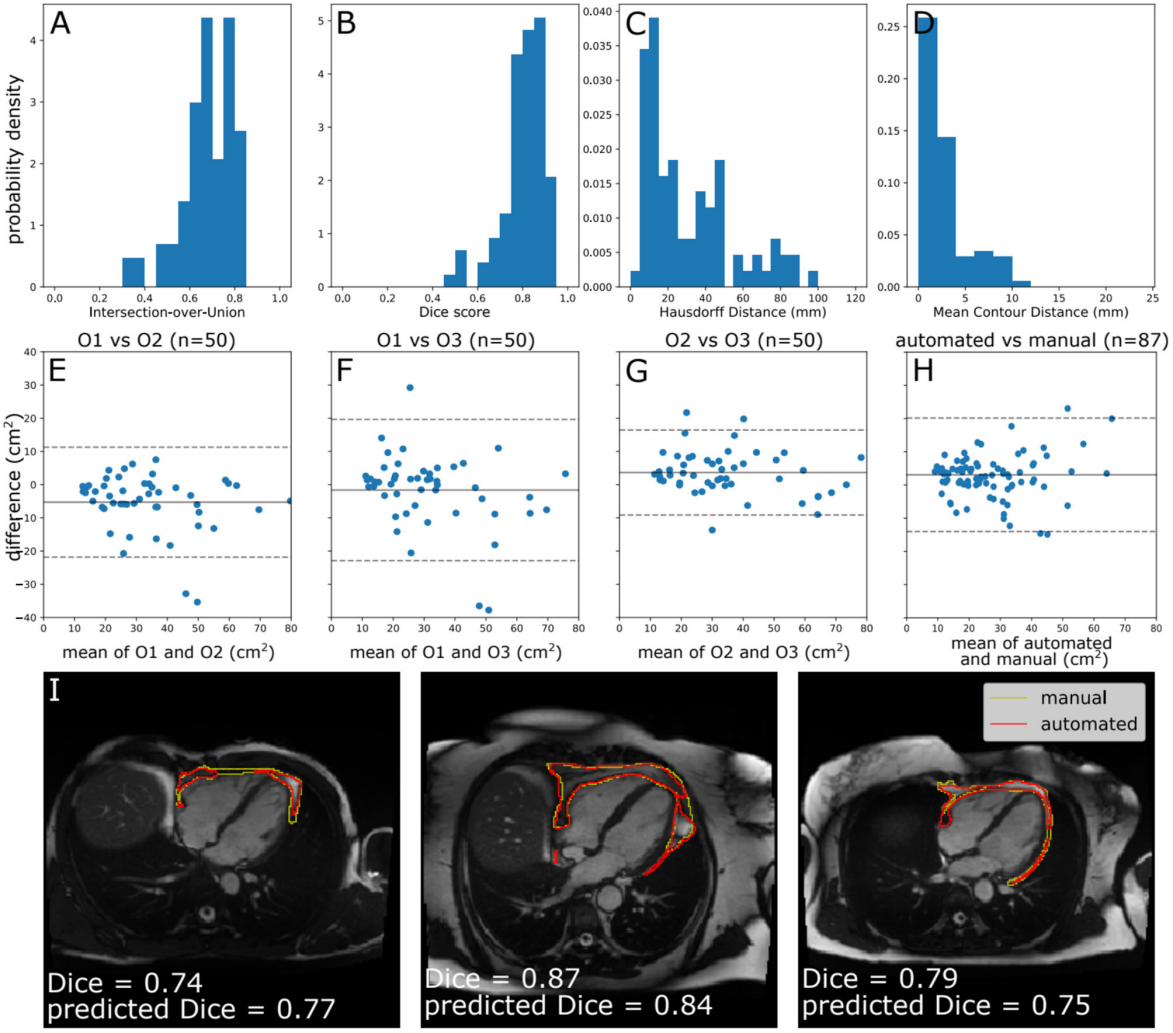
Model performance. **(A–D)** Histograms of standard segmentation performance metrics on the test set (*n* = 87). **(E–H)** Bland–Altman plots of PAT area between manual measurement between measurements by different human observers, and a human observer (O1) and automated measurement. The x-axis denotes the average of two measurements and the y-axis denotes the difference between them. The dark line is the mean difference, and the dashed lines show ±1.96 standard deviations from the mean. **(E–G)** show the inter-observer variability evaluated by three observers (O1–O3) on a randomly selected subset of the manually contoured training set (*n* = 50 subjects). **(H)** shows the agreement between automated and manual measurements in the manually contoured test set (*n* = 87 subjects). **(I)** Example segmentations from the test set, with annotations showing Dice score and the predicted Dice score. Images reproduced with permission of UK Biobank.

**Figure 4:**
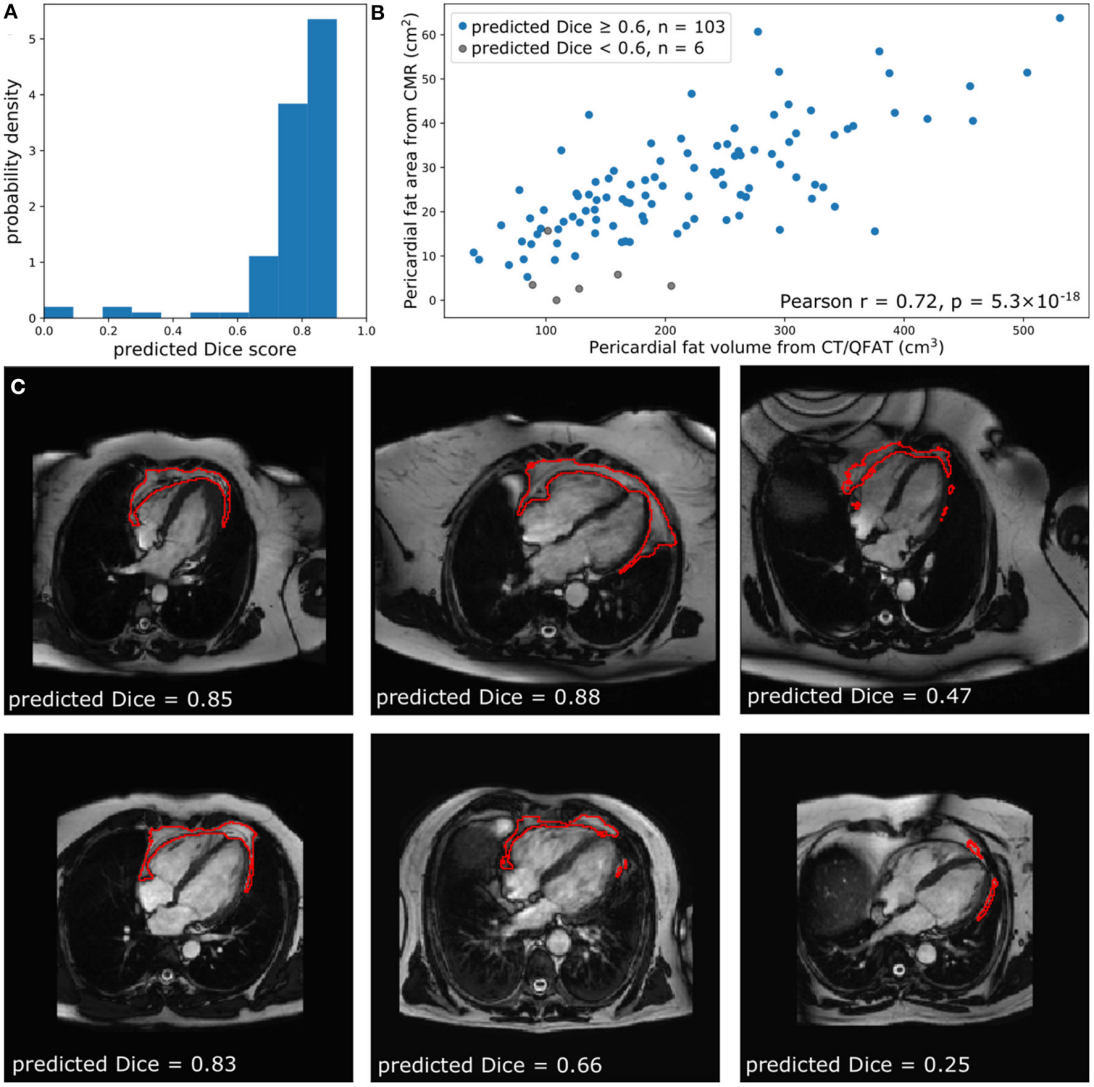
Comparison of quantified PAT from CT and CMR. **(A)** The predicted Dice scores of the segmented data. **(B)** Correlation between PAT volume quantified via QFAT software and PAT area quantified using our method. Subjects with a predicted Dice <0.6 were excluded from Pearson analysis. **(C)** Some example CMR images, their automatically segmented PAT, and the predicted segmentation quality are also shown for reference.

The performance of the automated segmentation within the test set relative to manual segmentations was good and very similar to the agreement between human observers (mean Dice score = 0.8). This was the case both for raw segmentation metrics (Table 1) and under Bland–Altman analysis (Figures 3E–H). Arguably, this is the best performance that may be achieved by an automated segmentation algorithm and reflects the inherently challenging nature of the PAT segmentation task. A few cases (*n* = 4, 4.5%) had poor segmentation quality (Dice score <0.6) (Figures 3A–D) and very large Hausdorff distances. This underlines the importance of the in-built QC feature, which would flag such cases. We also successfully applied the automated segmentation to the whole UKB imaging cohort (*n* = 45,519); 94.3% of cases (*n* = 42,928) had predicted Dice score of medium or good quality (mean predicted Dice score = 0.77). Example segmentation results from the UKB test dataset can be seen in Figure 3I. The automated segmentation also performed well in the external EVINCII dataset, with the majority of studies having medium/good segmentation quality (*n* = 103, 94.4%), with an overall mean predicted Dice score of 0.78 (Figure 4A). Running on a laptop PC with an Intel® Core™ i7-1165G7 processor, using a MC sample size (*N*) of 15, the model and QC step took 2.1 s, including image pre-processing and final estimation of Dice score.

**Table 1:** Standard segmentationperformance metrics for pairwise comparisons of manually contoured PAT by 3 observers (O1–O3), and comparing automated segmentation with manual for the test set.

**Metric**	**O1 vs. O2 (*n* = 50)**	**O1 vs. O3 (*n* = 50)**	**O2 vs. O3 (*n* = 50)**	**Automated vs. manual (O1) (*n* = 87)**
Intersection-over-Union	0.689 (0.133)	0.636 (0.153)	0.678 (0.123)	0.677 (0.116)
Dice	0.808 (0.102)	0.766 (0.127)	0.801 (0.096)	0.800 (0.090)
Mean contour distance (mm)	2.78 (2.44)	3.83 (3.48)	3.79 (3.44)	2.79 (2.35)
Hausdorff distance (mm)	30.1 (23.8)	37.0 (28.6)	39.9 (28.8)	29.9 (22.9)

*All values are mean (standard deviation)*.

### Correlation of Automated CMR PAT With CCT PAT Quantification

Within the EVINCII dataset, we tested the correlation of CMR PAT measures derived using our automated analysis with PAT derived using the QFAT tool from paired CCT scans. CMR studies with poor segmentation quality (predicted Dice score <0.6) were excluded from the analysis (*n* = 6). For illustration, we present example segmentations with a range of Dice scores in Figure 4C. There was a strong, statistically significant correlation between CCT PAT volume and CMR PAT area (Pearson *r* = 0.72, *p*-value 5.3 ×10^−18^, Figure 4B).

### Application to the UK Biobank Imaging Cohort and Association With Diabetes

We applied our neural network to CMR scans from 45,519 UKB participants. We excluded cases with a segmentation Dice score of <0.6 (*n* = 2,591, 5.7%). The remaining 42,928 participants were included in the analysis; of these, 2,529 were diabetic. Consistent with existing evidence (2), larger PAT area was associated with greater risk of diabetes in univariate and multivariable models (Table 2). In fully adjusted models, every 10 cm^2^ increase in PAT was associated with ~7% greater likelihood of diabetes independent of age, sex, and BMI.

**Table 2:** Logistic regression for prediction of diabetes in the UK Biobank dataset.

	**Variable**	**Odds ratio**	**95% CI**	***p*-value**
Model 1	PAT Area (cm^2^)	1.44	1.40, 1.47	8.43 ×10^−164^
Model 2	PAT Area (cm^2^)	1.36	1.32, 1.40	1.26 ×10^−92^
	Male sex	1.32	1.20, 1.44	8.97 ×10^−9^
	Age (years)	1.03	1.03, 1.04	4.66 ×10^−30^
Model 3	PAT Area (cm^2^)	1.07	1.03, 1.10	1.41 ×10^−4^
	Male sex	1.80	1.63, 1.98	2.43 ×10^−32^
	Age (years)	1.05	1.04, 1.05	7.91 ×10^−54^
	BMI	1.18	1.17, 1.19	4.27 ×10^−305^

*The dataset includes 2,529 diabetics and 40,399 non-diabetics. Odds ratios for PAT area are indicated for an increase of 10 cm^2^. BMI, body mass index; CI, confidence interval; PAT, pericardial adipose tissue*.

## Discussion

### Summary of Findings

We present a novel method for PAT quantification using standard-of-care CMR images, fully automated through a convolutional neural network with an in-built QC algorithm. The automated segmentation tool performed well within the test set, the whole UKB imaging cohort, and an external dataset, producing segmentation agreement close to that of human observers. Our segmentation method demonstrates validity against CCT PAT quantification, with a strong statistically significant correlation between paired CMR and CCT PAT measurements. Furthermore, we demonstrate, within the UKB, expected clinical association of CMR PAT with diabetes independent of age, sex, and BMI. Thus, the proposed CMR PAT method has great potential in facilitating investigation of the clinical significance of PAT in large population cohorts.

### Comparison With Existing Work

Limited studies have attempted to quantify and study the clinical associations of CMR PAT. In a study from 1992, Ross et al. (30) proposed a method for quantification of abdominal and subcutaneous fat on spin echo T1-weighted magnetic resonance imaging (MRI) sequences. They propose a signal intensity threshold for defining adipose tissue pixels; the area of adipose tissue regions was then calculated by summing adipose tissue pixels and multiplying by the pixel surface area; this area was then multiplied by slice thickness to derive the volume of adipose tissue. Unsupervised approaches for quantification of abdominal fat using this method have been developed using small datasets (31). More recently, these principles have been repurposed to derive CMR measures of thoracic fat using spin echo T1-weighted CMR acquisitions with zero slice gap and to investigate clinical associations in small cohorts (32–34). While this approach has had some utility, there are two fundamental limitations. Firstly, as the thresholding is based on pixel intensity levels, this is subject to variation based on technical (e.g., magnet strength, acquisition sequences, vendor) factors; as such, the threshold would need to be re-established depending on technical parameters. Secondly, because the methodology aims to derive a volumetric measure of adipose tissue, dedicated acquisitions with zero slice gap have been obtained. As standard protocols do not have zero slice gap (usually 5–8 mm slice gap), this approach, as it stands, is not suitable for application to standard-of-care CMR images.

Ding et al. (14) propose another approach for CMR PAT quantification; they present a limited study demonstrating feasibility of a fully automated pericardial fat quantification method from water/fat-resolved whole-heart non-contrast coronary magnetic resonance angiography. The very small sample size (*n* = 10) in this limited feasibility study precludes any meaningful assessment of model performance and the clinical validity of the proposed measurement is not known. Furthermore, as fat/water sequences are not routinely acquired as part of standard CMR studies, this methodology is unlikely to have wide application.

In a similar approach to our work, Rado et al. (15) quantify epicardial and pericardial fat areas from four-chamber cine images. They use a manual analysis protocol taking measurements in end-diastole and end-systole and making distinction between epicardial and pericardial fat area. They use their manual analysis measures (*n* = 374) to investigation associations with impaired glucose metabolism and left ventricular function. In developing our SOP, we also experimented with distinguishing between epicardial and pericardial fat areas. However, on inspection of a large number of studies, it became apparent that reliable distinction of these two areas was not possible for a substantial number of cases. Hence, we opted for a simpler approach of using a single en bloc contour. The strong correlation of our measure with CCT PAT quantification and observed associations with diabetes suggest that quantification according to our SOP does not detract from the potential utility of the measurement. Furthermore, the simplicity of our method enabled development of a fully automated analysis tool, which is essential for study of CMR PAT in large datasets.

### Technical Implications

In terms of the technical details of our neural network, the Multi-Residual U-net architecture (23) was vital, yielding far better results than “vanilla” U-nets (21) (data not shown). Meanwhile, a QC method has been demonstrated using an extension of a stochastic network, which approximates Bayesian MC sampling (24). Consistent with prior work, we find that measures of similarity between MC samples are correlated with segmentation quality; intuitively, this corresponds to how “sure” the network is of the output. However, in contrast, we found that the mean pairwise Dice score *d*^*MC*^ yielded best prediction, in contrast to the global intersection-over-union *IoU*^*G*^ used in previous work, and that an additional linear correction was required.

A potential consideration is whether better segmentation accuracy could be obtained *via* the removal of the stochastic component, thereby providing a single prediction. This would be undesirable for a number of reasons. Firstly, it is important to have some estimate of segmentation quality, which can only be provided if the actual segmentation is derived from our stochastic process. Secondly, a comparison with a non-deterministic MultiresUNet is provided in Supplementary Figure 1, and the accuracy is comparable with our stochastic model. However, note that dropout was not used within training of this network for the following reason: The MultiResUNet architecture makes extensive use of batch normalization. Because of a phenomenon known as variance shift, the combination of batch normalization and dropout often produces reduced accuracy once the dropout is “turned off” (35). However, this problem does not apply to our results, as the dropout is kept permanently active.

## Strengths and Limitations

Using a modest manually annotated dataset, we have achieved good segmentation accuracy, with a mean Dice score of 0.80. However, the performance of machine learning tools may be reduced when applied to external datasets (decline in generalizability); to minimize this effect, we made use of robust data augmentation procedures during training (26). We are reassured by the good performance of our tool on the whole UKB imaging cohort and on the external EVINCII CMR dataset. We use a very simplified SOP taking PAT area measurement from a single 2D slice; clearly, this approach does not accurately quantify the volume of mediastinal fat. However, we demonstrate correlation of our measurement with established volumetric CCT PAT measure and replicate known clinical associations with diabetes. This suggests that our CMR PAT measure is valid as a marker to study associations with the PAT exposure. Indeed, we would argue that complicated acquisitions to quantify thoracic fat have hampered previous attempts to make wide practical use of this measure. A potential limitation of the method is that the model cannot distinguish between specific pathologies—e.g., fat or fluid. In UKB, and similar cohorts, we do not expect this to be a significant source of error, as there are very few participants with pericardial effusions. However, with broader application of the tool to clinical cohorts, such considerations may be more relevant. Further studies in large cohorts are now needed to establish the clinical utility of this CMR PAT measure in different settings and patient cohorts, and the proposed automated tool will facilitate such studies in large (and small) cohorts. As we use standard-of-care images, the CMR PAT measurement can be retrospectively applied to any existing dataset and, furthermore, if clinical value of this metric is established, it could be readily integrated into clinical practice.

## Conclusion

We present a novel fully automated quality-controlled method for CMR PAT quantification using standard-of-care four-chamber cine images. Throughout the study, we demonstrate that our QC method functions as intended, and we demonstrate that the segmentation performance of this method is equivalent to inter-observer variability and that the area extracted by our method is strongly correlated with measurements taken using reference standard CCT quantification. Finally, we demonstrate that our CMR PAT quantification method can recapitulate known clinical associations with diabetes. Overall, we present a novel tool that is now ready to be used for new research.

## Data Availability Statement

Publicly available datasets were analyzed in this study. This data can be found here: this project made use of data from UKB. Data access was granted through access application 2964. All derived data including pericardial fat area values and image segmentations will be returned to UKB, as per standard UKB data returns policy. Access to these data may be obtained by bone fide researchers through a formal application process. More information on data access procedures may be found through the UKB website: https://www.ukbiobank.ac.uk.

## Ethics Statement

The studies involving human participants were reviewed and approved by UKB studies from the NHS National Research Ethics Service on 17th June 2011 (Ref 11/NW/0382) and extended on 10th May 2016 (Ref 16/NW/0274). Use of paired CT and CMR data from the EVINCI study was covered by a Data Protection Impact Assessment by the Data Protection Officer of Barts Health NHS Trust. For the original EVINCII study, local ethical approval was provided (REC Number: 10/H0721/79) and all subjects gave written informed consent. The patients/participants provided their written informed consent to participate in this study.

## Author Contributions

SEP, ZR-E, and MA conceived the idea, developed the contouring method, and contributed to manual analysis. AB led on the machine learning methodology, the main manual analysis of CMR data, and image analysis of cardiac CT data. ZR-E advised on statistical analysis. AB and ZR-E wrote the manuscript. AML advised on technical methods. FP and DD advised on cardiac CT validation. DD advised on analysis of cardiac CT. NCH and SEP provided overall supervision. All authors contributed to drafting the final manuscript and provided critical feedback.

## Conflict of Interest

The intellectual property for the code presented in this paper belongs to Barts Health and not the authors.
