# Supplementary material for: Automated Quality-Controlled Cardiovascular Magnetic Resonance Pericardial Fat Quantification Using a Convolutional Neural Network in the UK Biobank
Source: Front Cardiovasc Med. 2021 Jul 7;8:677574. doi: 10.3389/fcvm.2021.677574 (PMC8294033; doi:10.3389/fcvm.2021.677574)
Supplement: Supplementary file 1 [file Data_Sheet_1.docx]

**Supplementary Material**

**Selection of Monte Carlo sample size**

It is expected that drawing more samples from a well-trained network will increase its accuracy, but with diminishing returns. Where the area of “foreground” pixels is particularly of interest (as in this use case, quantifying the area of PAT), we can report the mean and standard deviation of the areas across the $N$ samples. The standard deviation is useful in that it can be propagated forward and used in downstream analyses. Again, we would expect increasing $N$ to increase this standard deviation for any individual input image, but with diminishing returns.

Thus, we examined the effect of changing $N$ on the mean segmentation accuracy and the mean estimated standard deviation of segmented areas (**Figure S1)**. Where comparisons with a deterministic neural network are shown (**Figure S1A**), this network was trained separately, using the same split data for training and evaluation - consistent with prior work(24), there was no sacrifice in segmentation quality by using a stochastic method. There is a clear trade-off between computational resources and accuracy - when $N$ is increased (requiring more computational time), there is an increase in the segmentation accuracy of the consensus, the uncertainty in the derived areas, and the correlations between accuracy proxies and the true accuracy. However, there are diminishing returns on all of these values. On this basis, $N$ was set to 15 for all further work.

**Selection of metric used in quality control**

When multiple MC samples are drawn from the stochastic neural network, their level of agreement is correlated with the quality of the consensus segmentation (Roy et al. 2019). However, their level of agreement can be measured in many different ways. Here, we use 4 metrics, based on the well-known intersection-over-union (or Jacard Index) and the Dice score. However, there are a number of ways to formulate these for a summary statistic over $N$ MC samples (denoted $S_{1}...S_{N}$).

The IoU can be calculated globally (as in (Roy et al. 2019)):

$$IoU^{G}=\frac{|\underset{i=1}{\overset{N}{\bigcap}}S_{i}|}{|\underset{i=1}{\overset{N}{\bigcup}}S_{i}|}$$

Alternatively, it can be calculated as the mean of pairwise combinations across the $N$ MC samples:

$$IoU^{MC}=\frac{2(N-2)}{N!}\frac{\sum_{i=1}^{N} \sum_{j=1}^{i-1} |S_{i}\cap S_{j}|}{|S_{i}\cup S_{j}|}$$

Likewise, the Dice score can be calculated globally over all MC samples:

$$d^{G}=\frac{N\times|\underset{i=1}{\overset{N}{\bigcap}}S_{i}|}{\sum_{i=1}^{N} |S_{i}|}$$

Or, it can be calculated as the mean of pairwise combinations (Roy et al. 2019):

$$d^{MC}=\frac{2(N-2)}{N!}\frac{\sum_{i=1}^{N} \sum_{j=1}^{i-1} 2|S_{i}\cap S_{j}|}{|S_{i}|+|S_{j}|}$$

The performance of these metrics can be quantified in a number of ways (after a linear correction is applied):

- The Pearson correlation coefficient between the prediction and the true Dice score, which is a measure of the accuracy with which a linear model can predict the true Dice score.
- The Mean Absolute Error (MAE) between the prediction (after linear correction) and the true Dice score – where a smaller value indicates a better prediction of the true Dice score
- How well the predicted categories of segmentation quality match up with those provided by the true Dice score, with descriptors of poor, medium and good corresponding to Dice scores of <0.6, 0.6-0.8 and ≥0.8 respectively.

These indicators of success can be seen in **Figure S2**, which also shows the linear corrections applied to each metric. Although the mean absolute error of predicted Dice scores is nearly identical (0.044 for both $d^{MC}$, and $IoU^{MC}$), there is a stronger linear relationship found for $d^{MC}$, and most importantly, an advantage in the fraction of correctly-classified examples in the test set. Based on these results, the best predictor of segmentation accuracy is the mean pairwise Dice score $d^{MC}$. This is contrary to a previous result(24), which finds the global IoU $IoU^{G}$ to be superior to $d^{MC}$, and additionally did not find a requirement for linear corrections. For the remainder of the paper we use $N=15$ for MC sampling, and the mean pairwise Dice score $d^{MC}$ with linear correction for predicting the Dice score of each individual segmentation.

**Supplementary Figure 1. Effects of** $\boldsymbol{N}$ **on model performance**


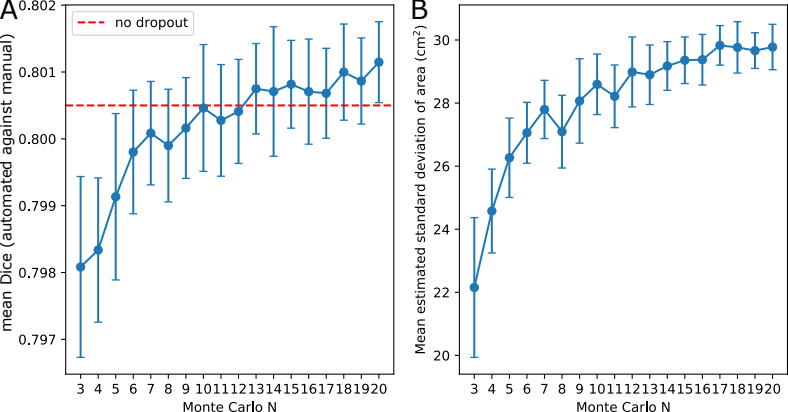


***Supplementary Figure 1:*** *Each graph here shows mean*$\pm$*standard deviation of 15 runs for each Monte Carlo* $N$*, over the test set (*$n=87$ *participants).* ***A.*** *Mean Dice score. For comparison, the Dice score obtained with a deterministic MultiResUNet is also shown (dotted line).* ***B.*** *Mean estimated standard deviation of PAT area.*

**Supplementary Figure 2. Effectiveness of different methods for predicting segmentation quality** for the test set ($n=87$), as well as statistics describing their performance (Pearson $r$, the mean absolute error of predictions after linear corrections, and the category accuracy).
